# Immune checkpoint knockout screen reveals TIGIT-CD155 interaction as a significant modulator of adapter CAR-T cell function in acute myeloid leukemia

**DOI:** 10.1080/2162402X.2026.2717853

**Published:** 2026-09-17

**Authors:** Anna-Sophia Mast, Daniel Atar, Lara Ruoff, Sophia Scheuermann, Beate Kristmann, Peter Lang, Christian Martin Seitz, Patrick Schlegel

**Affiliations:** a Department of Hematology, Oncology, Gastroenterology, Nephrology, Rheumatology, University Children’s Hospital Tuebingen, Tuebingen, Germany; b iFIT Cluster of Excellence EXC 2180 ‘Image-Guided and Functionally Instructed Tumor Therapies’, University of Tuebingen, Tuebingen, Germany; c Hopp-Children's Cancer Center Heidelberg (KiTZ), Heidelberg, Germany; d Clinical Cooperation Unit Pediatric Oncology, German Cancer Research Center (DKFZ), Germany; e Department of Pediatric Oncology, Hematology, and Immunology, Heidelberg University Hospital, Heidelberg, Germany; f Department of Pediatric Hematology, Oncology and Stem Cell Transplantation, University of Regensburg, Regensburg, Germany; g Leibniz Institute for Immunotherapy, Regensburg, Germany

**Keywords:** Adapter CAR T cell therapy, AML, immune checkpoint receptor

## Abstract

Chimeric antigen receptor (CAR) T cell therapy for acute myeloid leukemia (AML) faces critical challenges, including severe on-target off-tumor toxicities, antigen heterogeneity, and primary immune evasion mechanisms. To address these limitations, we previously developed an adapter CAR (AdCAR) platform that enables transient and combinatorial targeting to improve the safety and efficacy of CAR-T-cell therapy in AML. In this study, we further optimized the AdCAR system by investigating the role of immune checkpoint receptors (ICRs), particularly PD-1, CD96, LAG-3, TIM-3, and TIGIT, as well as the immunomodulatory molecule CD276, in suppressing AdCAR-T-cell function. Using multiparametric flow cytometry, we analyzed the expression of immune checkpoint ligands (ICLs) across multiple cancer entities, including three well-characterized AML cell lines, primary AML bone marrow samples, and healthy bone marrow as a reference. We then examined the inducibility of ICLs on AML cells after AdCAR-T-cell engagement. To evaluate the functional impact of immune checkpoint inhibition (ICI), we generated AdCAR-T-cells with CRISPR/Cas9-mediated knockouts (KOs) of PD-1, CD96, CD276, LAG-3, TIM-3, or TIGIT, and analyzed their cytotoxic potential in vitro. In our setting, PD-1 disruption did not significantly enhance AdCAR-T cytotoxicity against MOLM-13 wildtype, while TIGIT KO significantly enhanced AdCAR-T cytotoxicity specifically across five leukemia- and lymphoma-cell lines. Notably, pharmacological PD-1 blockade resulted in greater cytotoxicity than PD-1 gene disruption, whereas TIGIT blockade showed comparable effects to TIGIT KO. Our study identified TIGIT KO as a potent enhancer of AdCAR-T-cell function and supports ICR gene disruption as a promising approach to improve therapeutic efficacy while reducing the systemic toxicities commonly associated with ICI therapy.

## Background

Chimeric antigen receptor (CAR) T cell therapy has substantially improved overall survival in patients with recurrent or refractory B-lineage malignancies. One key factor in the recent success of CAR-T cell therapy in conditions such as B-cell acute lymphoblastic leukemia (B-ALL), B-cell non-Hodgkin lymphoma (B-NHL), and multiple myeloma has been the identification of target antigens that are predominantly expressed on non-essential tissues within the B-lineage compartment.^1-3^ In acute myeloid leukemia (AML), CAR-T cell therapies targeting antigens such as CD33, CD38, and CD123 have shown promising efficacy in both preclinical and clinical settings.^4-10^ However, their clinical application remains limited by the lack of leukemia-specific surface markers. Most target antigens are also expressed on healthy myeloid cells and hematopoietic progenitor cells (HPCs), resulting in severe on-target off-tumor toxicities.^11^ These toxicities can lead to the depletion of bone marrow-derived HPCs, ultimately causing myeloid aplasia and bone marrow dysfunction.^12-15^ To mitigate these toxicities, conventional autologous CAR-T cells have been successfully employed as remission-induction therapy in patients with relapsed or refractory AML prior to undergoing allogeneic hematopoietic stem cell transplantation (alloHSCT). Notably, this approach has required CAR-T cell doses tenfold higher than those used in CD19-directed CAR-T cell therapy for B-ALL to achieve sustained remission.^16^ ^,^ ^17^ In contrast to CD19-directed CAR-T cell therapy, the complete elimination of AML-targeted CAR-T cells through the conditioning regimen is critical for the restoration of myelopoiesis following alloHSCT and, thereby, for patient recovery.^17^ ^,^ ^18^ Alternative strategies to overcome on-target off-tumor toxicity in AML include genetic modification or knockout (KO) of the target antigen in hematopoietic stem cells (HSCs), which are co-infused to rescue hematopoiesis and compensate for the loss of reconstituting HPCs.^19^ ^,^ ^20^

Additional major challenges in CAR-T cell therapy for AML include intratumoral heterogeneity and the emergence of antigen escape variants. These limitations emphasize the need for multitargeted CAR-T cell strategies to achieve durable disease control while minimizing treatment-related toxicities.^11^ ^,^ ^15^ ^,^ ^21^ To address these challenges, we developed the adapter CAR (AdCAR) technology, a modular system for flexible antigen targeting. AdCAR-T cells recognize a defined linker-label-epitope (LLE) incorporated into biotinylated adapter molecules (AMs). These AMs engage with specific target antigens, enabling antigen-specific T-cell activation (Figure S1). The AdCAR platform has previously demonstrated efficacy and is particularly well-suited for AML, as it allows both combinatorial and transient targeting.^22^ ^,^ ^23^

Another major obstacle limiting the success of cancer immunotherapy in AML is the immunosuppressive tumor microenvironment (TME).^24^ ^,^ ^25^ In malignancies with a high mutational burden, such as melanoma, non-small-cell lung cancer, and mismatch repair-deficient colorectal cancer, antibody-based immune checkpoint inhibition (ICI) has led to significantly improved disease control and overall survival.^26-32^ The efficacy of ICI strongly correlates with mutational burden.^26^ A significant clinical benefit has been observed with PD-1 and/or CTLA-4 blocking antibodies in melanoma, harboring a high mutational burden and an upregulation of ICR pathways.^33^ ^,^ ^34^ By contrast, AMLs rank among the cancers with the lowest mutational burden and display 10-100 times fewer mutations per individual cancer compared to skin cancers, which correlates with modest and inconsistent responses to ICI.^26^ ^,^ ^34^ ^,^ ^35^ Recently, the immune checkpoint receptors (ICR) TIM-3, LAG-3, and TIGIT have been identified to substantially inhibit T cell anticancer immunity, which has led to the development of novel clinical antibodies targeting these ICR-related pathways.^36^ A critical factor in harnessing the potency of ICI is the availability of tumor-responsive immune cells. The composition of immune cells within tumor lesions is shaped by ICI therapy, as observed in colorectal cancer patients.^37^ Unlike conventional T cells, CAR-T cells do not depend on T-cell receptor (TCR)-mediated antigen recognition, enabling effective tumor recognition even in tumors with low mutational burden.^38^ However, (CAR) T cells’ tumor interaction induces the upregulation of ICRs within the TME, leading to a functional impairment of immune cells.^39^ ^,^ ^40^ Notably, this immunosuppressive state can sensitize immune cells to ICI, enhancing their potential for functional reactivation.^26^

We demonstrate that CRISPR/Cas9-mediated inactivation of ICR genes in AdCAR-T cells enhances their antileukemic activity in AML by specifically eliminating inhibitory signaling within the genetically modified cells. This approach provides a distinct advantage over antibody-based ICR blockade, which is often associated with systemic adverse events, including the onset of hyperinflammatory autoimmune responses.^37^

## Methods

### Cell lines and culturing conditions

All cell lines were purchased from ATCC or DSMZ and maintained according to the manufacturer’s instructions at 37 °C, 95% humidity, and 5% CO_2_ in complete media [RPMI 1640 or DMEM (Hs578T) supplemented with 10% or 20% heat-inactivated fetal bovine serum (FBS, ThermoFisher Scientific), 2 mM L-glutamine, 1 mM sodium pyruvate (Sigma–Aldrich®), 100 units/mL of penicillin, and 100  µg/mL of streptomycin (Sigma–Aldrich®). Cells were stably transduced with lentivirus to express firefly luciferase as a reporter. PDL1 and CD155 were overexpressed as full-length human proteins via stable lentiviral transduction.

### Primary pediatric AML samples

Bone marrow samples were collected only during routine bone marrow aspirations from pediatric AML patients at the time of initial diagnosis or relapse, as well as from healthy donors, following written informed consent obtained from their caregivers. This study was designed and conducted in accordance with the principles of the Declaration of Helsinki and was approved by the local Institutional Review Board (819/2017BO1, 674/2017BO2).

### Immunophenotyping of pediatric AML samples

Immunophenotyping of primary pediatric AML and healthy donor bone marrow samples was conducted using a Cytek Aurora 5-laser spectral flow cytometer (Cytek Biosciences).

### Isolation of PBMCs and T cells

Whole blood from healthy volunteer donors was collected at the University Children's Hospital Tübingen under an institutional review board-approved exempt protocol (Approval No. 761/2015BO2) [study period 11/2019 to 08/2024]. Peripheral blood mononuclear cells (PBMCs) were isolated using a Ficoll-Paque density gradient centrifugation (Biocoll, Sigma–Aldrich®) according to the manufacturer’s instructions. T cells were subsequently isolated via magnetic cell separation using anti-CD4/CD8 microbeads (Miltenyi Biotec) according to the manufacturer’s instructions.

### Transduction and culture of T cells

Isolated T cells were cultured in TexMACS™ medium supplemented with 10 ng/mL IL-7 and 5 ng/mL IL-15 (Miltenyi Biotec) and activated using the human T-cell activating CD3/CD28 polymer matrix TransAct™ (1:100 dilution, Miltenyi Biotec). Lentiviral transduction was performed on day 3 after activation at a multiplicity of infection (MOI) of 3–10. Transduction efficacy was assessed on day 7 by flow cytometry, using an LLE-biotin-PE conjugate as the CAR detection reagent (Miltenyi Biotec). T cells were maintained in supplemented TexMACS™ medium at a concentration of 0.5–1 × 10^6^ cells/mL (Table S1). Experiments were conducted on days 12–14 after CD3/CD28 activation. ICR was knocked out via CRISPR/Cas9 (supplement).

### Manufacturing of antibodies and LC–LC–NHS biotinylation

Antibodies were expressed transiently in ExpiCHO according to the manufacturer’s instructions. The Biotinylation was conducted according to the manufacturer’s instructions of LC–LC–NHS biotin. Please find a detailed description in the supplementary methods (additional information in the supplement).

### Flow cytometry

Flow cytometry analyses were conducted using a FACSCanto™ II flow cytometer (Becton Dickinson) unless otherwise specified. Subsequent data analysis was conducted using FlowJo 10.8 software. The Mean Fluorescence Intensity Ratio (MFIR) was determined by dividing the median fluorescence intensity of the sample by that of the unstained control. MFIR-values >2 were defined as positive.

### ICL screening on tumor cell lines

Cell lines were cultured following ATCC recommendations. Antibody staining was performed according to the standard operating procedure at 4 °C in CliniMACS® PBS/EDTA buffer (Miltenyi Biotec). Tumor cells (Table S1) were stained with primary labeled antibodies, as listed in Table S2. Their antigen positivity was determined by comparison with unstained controls.

### ICL kinetics on AML cell lines in response to stimulation

The modulation of ICL expression levels in the AML cell lines MOLM-13, HL-60, and U937 in response to immune effector cell stimulation was assessed at 0, 48, and 72 h under co-culture conditions with AdCAR-T cells at an effector to target (E:T) ratio of 1:5. Specifically, 50,000 AdCAR-T cells (approximately 50% CAR expression) were co-incubated with 250,000 tumor cells in the presence of the LLE-CD33 monoclonal antibody (mAb) at a concentration of 10 ng/mL.

### ICR kinetics on AdCAR-T and KO variants in response to stimulation

The modulation of ICR expression in AdCAR-T cells was evaluated in response to co-incubation with Raji cells at an E:T ratio of 1:2. The AdCAR-T cell products included: (i) AdCAR-T^RNP Mock^, (ii) AdCAR-T^CD96 KO^, (iii) AdCAR-T^PD-1 KO^, (iv) AdCAR-T^LAG-3 KO^, (v) AdCAR-T^TIM-3 KO^, and (vi) AdCAR-T^TIGIT-KO^, all generated using CRISPR/Cas9 (Table S3). Specifically, 50,000 AdCAR-T cells were mixed with 100,000 Raji cells in the presence of 10 ng/ml LLE-CD19 mAb and analyzed at 0, 48, and 72 h.

### Immunophenotyping of primary bone marrow samples

This study was designed and conducted in accordance with the principles of the Declaration of Helsinki and was approved by the local Institutional Review Board (819/2017BO1, 674/2017BO2) [study period 01/2022 to 01/2023]. In brief, cryopreserved bone marrow samples were thawed, treated with DNase 1 (ThermoFisher Scientific), and incubated with a human Fc receptor (FcR) blocking reagent (Miltenyi Biotec). The antibodies, fluorochromes, and dilution factors used in the study are summarized in Table S2. Patient samples were acquired and unmixed using a Cytek Aurora 5-laser spectral flow cytometer (Cytek Biosciences) with the ‘Cytek Assay Settings’ in SpectroFlo® V3.0.3 software. The gating strategy is shown in Figure S3. Subsequent data analysis was performed using FlowJo 10.8 software. Antigen positivity was evaluated by comparison with fluorescence minus one (FMO) controls. The MFIR was calculated by dividing the MFI of the sample by that of the corresponding FMO control.

### Statistical analysis

Statistical analyses were performed separately for each target cell line, E:T ratio, and time point. Measurements across different E:T ratios and time points were not pooled for inferential testing. Technical replicates were summarized within each biological replicate, and *n* refers to the number of independent donors. Comparisons involving more than two conditions within a fixed experimental setting were analyzed using one-way ANOVA with Tukey’s post hoc test. Two-group comparisons were analyzed separately as two-group comparisons. A *p*-value of <0.05 was considered statistically significant. All statistical analyses were conducted using GraphPad Prism software (version 9.4.1).

## Results

### ICL expression heterogeneity across different malignancies

**Figure 1.**
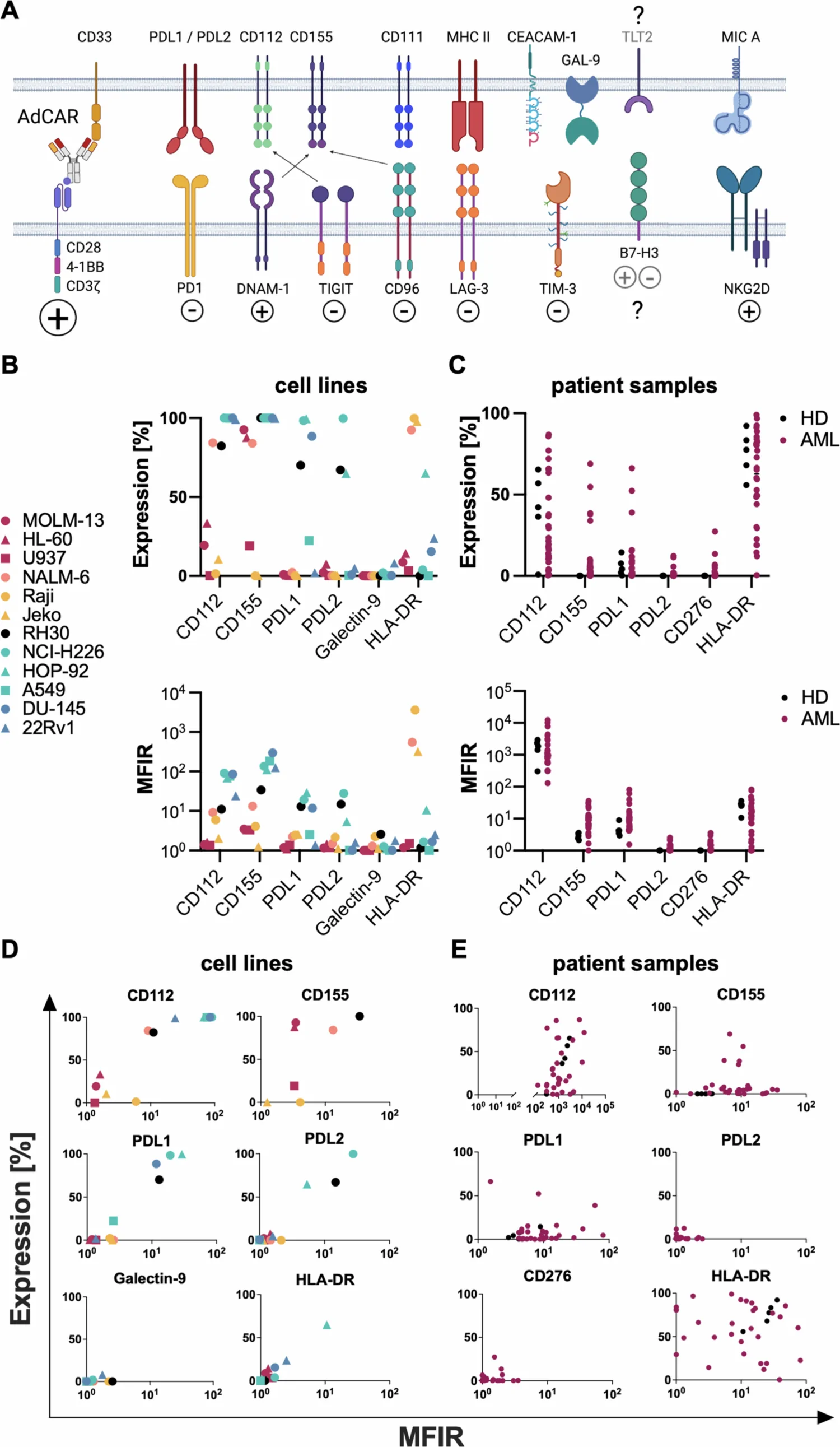
Expression and MFIR of ICLs on cancer cell lines and primary pediatric AML samples. (A) Schematic illustration of the interaction of AdCAR-T target recognition, mediated by an LLE-mAb and key immunomodulatory receptors, including ICRs, like PD-1 or TIGIT, and prominent activating receptors such as DNAM-1 and NKG2D. (B) Expression [%] and expression density [MFIR] of ICLs CD112, CD155, PDL1, PDL2, galectin-9, and HLA-DR on cancer cell lines (*n* = 12) analyzed by flow cytometry. MFIR-values >2 were defined as positive. Each data point represents a single measurement per cell line. Colors indicate distinct tumor entities (*n* = 6). (C) Expression [%] and expression density [MFIR] of ICLs CD112, CD155, PDL1, PDL2, CD276, and HLA-DR on primary pediatric AML samples (AML, *n* = 29) compared to healthy bone marrow donors (HD, *n* = 5). Each data point represents an individual patient or healthy donor. (D) Combined illustration of expression frequency and MFIR for each ICL across the analyzed cancer cell lines, with tumor entity color coding maintained. (E) Combined illustration of expression frequency and MFIR for each ICL across primary pediatric AML samples and healthy bone marrow donors. ICL expression was evaluated by flow cytometry using unstained controls for cancer cell lines, and FMO controls for primary samples; MFIR values were calculated relative to the respective controls. Abbreviations: MFIR: mean fluorescence intensity ratio; ICL: immune checkpoint ligand; AML: acute myeloid leukemia; HD: healthy donor; AdCAR: Adapter Chimeric Antigen Receptor; ICR: immune checkpoint receptor. FMO: Fluorescent minus one control. Statistical comparisons were performed using the Mann-Whitney test, with *p*-values <  0.05 considered statistically significant. Only statistically significant differences are indicated. *p < 0.05; **p < 0.01; ***p < 0.001.

To characterize the immunoregulatory landscape across different malignancies, we analyzed the surface expression of selected ICLs on 12 tumor cell lines representing six cancer entities. Specifically, we analyzed the surface expression of CD112 and CD155 (both ligands for TIGIT), CD274 (PDL1) and CD273 (PDL2) (both ligands for CD279 (PD-1)), galectin-9 (ligand for TIM-3), and HLA-DR (ligand for LAG-3) (Figure 1A). The AML cell lines MOLM-13, HL-60, and U937 exhibited overall low ICL expression, both in terms of frequency of positive cells and surface density (Figure 1B, D). CD155 was detected in more than 80% of MOLM-13 and HL-60 cells but only in 20% of U937 cells, with moderate expression levels per cell (MFIR ≈ 3). In contrast, solid tumor cell lines (including lung and prostate carcinomas as well as rhabdomyosarcoma) showed high expression frequencies of CD112, CD155, PDL1, and PDL2. CD112 and CD155 displayed high surface density (MFIR 10–300), whereas PDL1 expression was more heterogeneous (MFIR 1–30). Notably, PDL1 and PDL2 were exclusively expressed in solid tumor lines. As expected, the B-lineage-derived cancer cell lines NALM-6, Raji, and JeKo-1 demonstrated high expression frequency and density of HLA-DR. Galectin-9 was not significantly expressed in any of the tested cell lines, and HLA-DR expression remained low in non-hematologic lines. Our findings indicate heterogeneous expression patterns of ICLs, both within individual cancer entities and across different malignancies.

### Immune checkpoint ligand expression heterogeneity in a cohort of 29 primary pediatric AML samples

To further investigate the clinical relevance of these findings, we next analyzed the expression of ICLs in primary pediatric AML samples (*n* = 29) using multiparametric flow cytometry. The cohort included representative samples obtained at initial diagnosis (*n* = 20) and relapse (*n* = 9). Due to the limited number of samples, the data are representative but remain descriptive. The clinical and biological characteristics of the analyzed AML samples are provided in Table S4. We assessed the expression of both immunophenotypic markers, including CD45, CD3, CD34, CD33, CD38, CD123, CD135, CD371, and IL1RAP, as well as ICLs, specifically CD112, CD155, PDL1, PDL2, CD276, and HLA-DR. Leukemic blasts were identified within the CD45-intermediate/SSC-low population as well as based on their characteristic immunophenotypic profile and were compared with the corresponding CD45-intermediate/SSC-low population of healthy bone marrow controls, representing the physiological hematopoietic progenitor compartment (Figure S3). Overall, ICL expression was highly heterogeneous across individual patient samples (Figure 1C, E). CD112 showed the highest expression frequency and per-cell density, both in AML blasts and in the physiological CD45 intermediate population of healthy bone marrow controls. CD155 was detected at relevant levels in 17% (5/29) of samples. PDL1 expression was limited to a small subset of AMLs, whereas PDL2 and CD276 were expressed at negligible levels. Although HLA-DR was frequently detected, it showed substantial inter-patient variability in both frequency and surface density. Compared with the corresponding physiological CD45-intermediate population of healthy bone marrow controls, AML blasts exhibited significantly higher expression of CD155 and PDL1, whereas the majority of other ICL expression levels did not differ significantly between AML samples and healthy controls. Likewise, no significant differences in ICL expression were observed between samples obtained at initial diagnosis and those collected at relapse (Figure S4A). The frequency of T-cell infiltration in the bone marrow was comparable between AML patients and healthy donors (Figure S4B), and in our dataset, no association was found between ICL expression and the degree of T-cell infiltration (Figure S4C). In summary, ICL expression in pediatric AML is highly heterogeneous. CD112 and HLA-DR are the most consistently expressed ligands, whereas CD155, PDL1, PDL2, and CD276 exhibit variable and often low expression levels.

### CAR targeting induces dynamic alterations in ICL expression in AML cell lines

**Figure 2.**
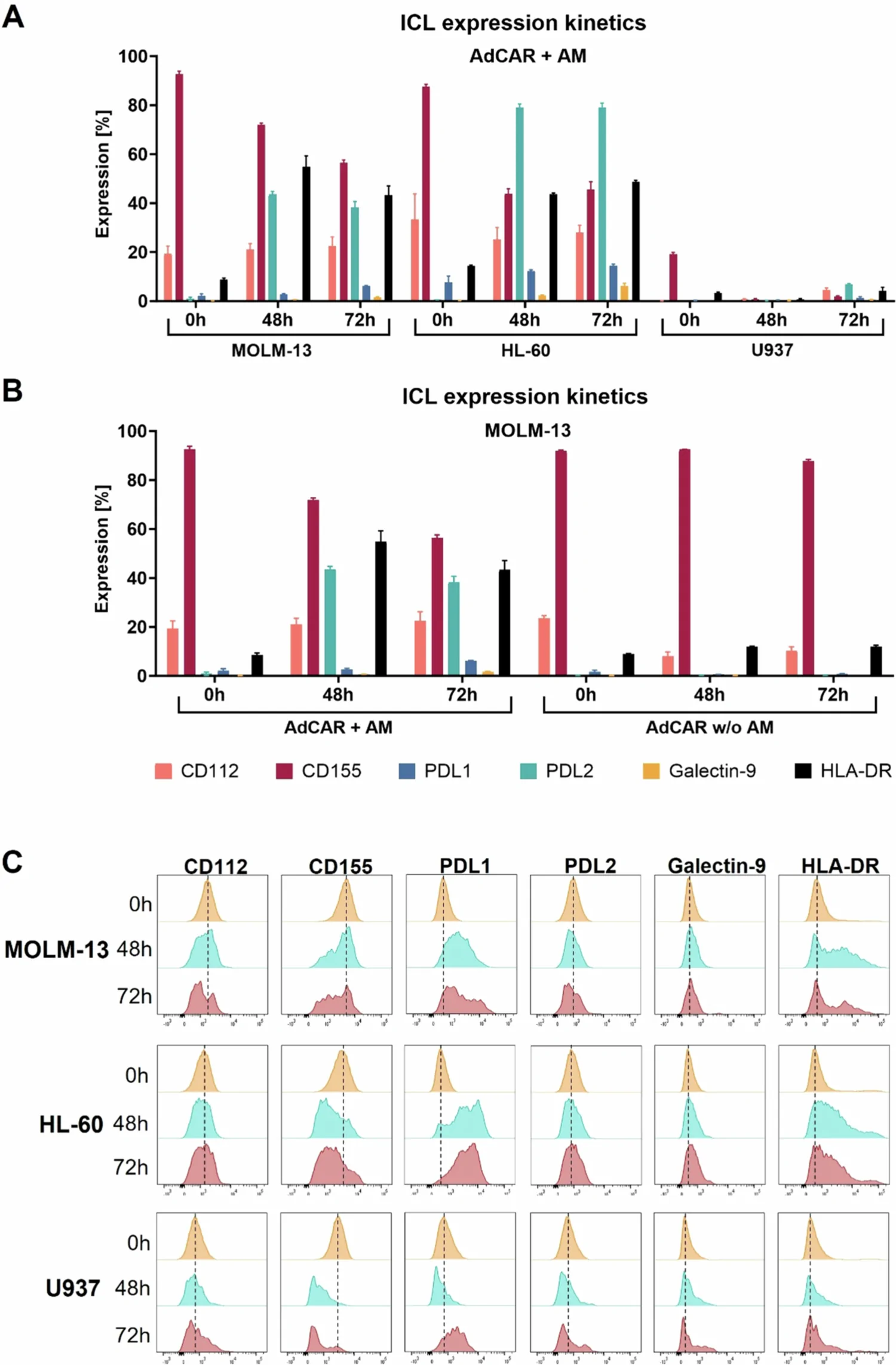
Impact of AdCAR-T cell targeting on the expression kinetics of ICLs in AML cell lines. (A) Kinetics of Immune Checkpoint Ligand expression (CD112, CD155, PDL1, PDL2, galectin-9, HLA-DR) in AML cell lines (MOLM-13, HL-60, and U937) co-cultured with AdCAR-T cells at an E:T ratio of 1:5 in the presence of 10 ng/mL LLE-CD33 mAb. Expression levels [%] were assessed at 0, 48, and 72 h by flow cytometry. *n* = 3, with error bars indicating the SEM. (B) ICL expression on MOLM-13 co-cultured with AdCAR-T cells at an E:T ratio of 1:5, both with and without 10 ng/ml LLE-CD33 mAb at the indicated time points. *n* = 3, error bars indicate the SEM. (C) Representative histograms showing the fluorescence intensity profiles of ICLs on AML cell lines after co-culture with AdCAR-T cells at an E:T ratio of 1:5 in the presence of 10 ng/mL LLE-CD33 mAb at 0, 48, and 72 h. Abbreviations: AM: adapter molecule; E:T: effector-to-target ratio; SEM: standard error of the mean.

To assess the impact of AdCAR-T cell activity on ICL expression in AML, MOLM-13, HL-60, and U937 cell lines were co-cultured with AdCAR-T cells in the presence or absence of LLE-CD33 mAb for 72 h (Figure 2). In MOLM-13 and HL-60 cells, CD33-directed AdCAR-T cell engagement led to upregulation of PDL1 and HLA-DR, downregulation of CD155, and no detectable changes in CD112, PDL2, or galectin-9 surface expression (Figure 2A, C). ICL expression in U937 cells remained unchanged under all conditions. Notably, these expression changes were specifically associated with AdCAR-T cell activation and antigen engagement, as they were only observed in the presence of both AdCAR-T cells and the LLE-CD33 adapter. No such modulation occurred in the absence of the adapter molecule (Figure 2B).

### Optimization of the ICR KO protocol for the generation of AdCAR-T cells

**Figure 3.**
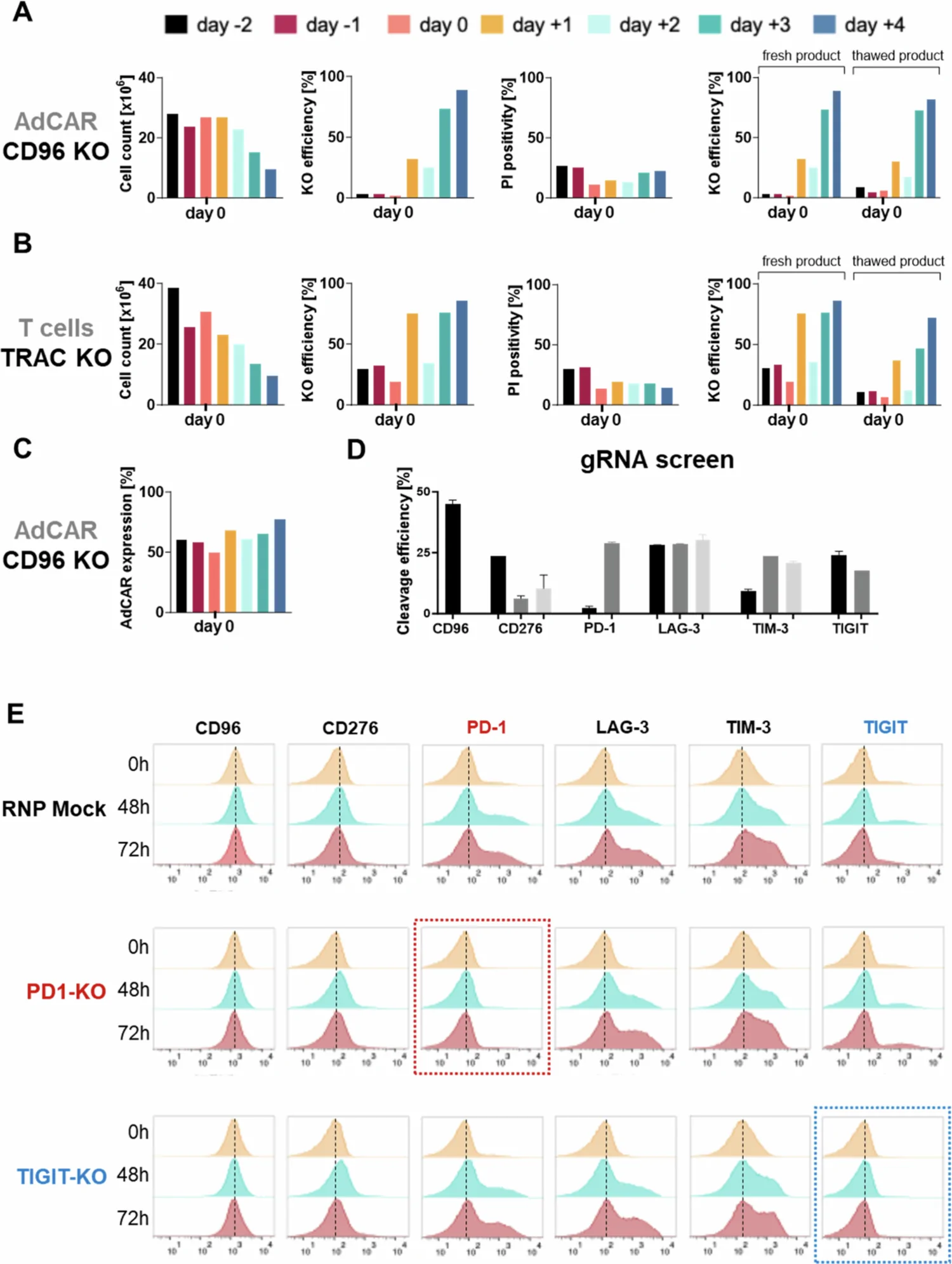
Optimization of CRISPR/Cas9-mediated gene editing in AdCAR-T cells and characterization of ICR expression kinetics on AdCAR-T cells during co-culture with tumor cells. To determine the optimal time point for CRISPR/Cas9-mediated ICR KO within the CAR-T cell expansion protocol, CD96 (in AdCAR-T cells, A) and T cell receptor *α* chain (*TRAC*, in primary T cells, B) were knocked out by electroporation between day −2 and day +4, relative to CD3/CD28 TransAct-mediated activation (day 0). Conditions were evaluated based on cell expansion (total cell count), viability (propidium iodide [PI] negativity), and KO efficiency on day +10 post-activation, including post-cryopreservation analysis to evaluate product stability. *n* = 1. (C) Analysis of AdCAR expression on day +10 after CD3/CD28 activation relative to the timing of CRISPR/Cas9 electroporation. (D) Screening of gRNAs targeting CD96, CD276, PD-1, LAG-3, TIM-3, and TIGIT in primary T cells. KO efficiency was assessed using the GeneArt Genomic Cleavage Detection Kit (Thermo Fisher Scientific) by measuring genomic cleavage efficiency. Individual gRNAs are shown in varying shades of gray. *n* = 2 donors, with error bars indicating the SEM. (E) Representative histograms showing the fluorescence intensity profiles of ICRs on AdCAR-T cells following co-culture with Raji cells at an E:T ratio of 1:2 in the presence of 10 ng/mL LLE-CD19 mAb. Expression was assessed at 0, 48, and 72 h post-co-culture by flow cytometry. *n* refers to independent donors. Abbreviations: KO: knockout; ICR: immune checkpoint receptor; RNP: ribonucleoprotein; PI: propidium iodide; SEM: standard error of the mean; E:T: effector-to-target ratio.

Given the critical role of ICLs in modulating immunotherapy outcomes, we hypothesized that genetic inactivation of ICRs in AdCAR-T cells using CRISPR/Cas9 could enhance their anti-leukemic efficacy. To identify the optimal timing for CRISPR/Cas9-mediated KO within the CAR-T cell expansion protocol, the constitutively expressed protein CD96 (Figure 3A) and the T-cell receptor alpha chain (TRAC) (Figure 3B) were selected as representative targets. KOs were performed in AdCAR-T cells and primary T cells from day −2 to day +4, with CD3/CD28 TransAct-mediated T cell activation defined as day 0. Conditions were evaluated based on cell expansion (cell count), viability (propidium iodide positivity), and KO efficiency on day +10, as well as KO efficiency following cryopreservation and thawing of cells. Electroporation on days +3 and +4 resulted in reduced cell counts but achieved high KO-efficiencies in both CD96-KO and TRAC-KO conditions. On day +4, KO efficiencies reached 89% for CD96 and 86% for TRAC. Cryopreservation negatively affected the recovery of TRAC-KO cells, whereas CD96-KO cells remained largely unaffected, indicating that sensitivity to freeze-thaw stress may vary depending on the specific gene disrupted. CAR expression in CD96-KO AdCAR-T cells remained stable across all time points tested (Figure 3C). Based on the collective results, day +3 post-activation was determined to be the optimal time point for gene KO, balancing high KO efficiency while maintaining cell fitness and proliferative capacity. Gene disruption of ICRs was performed using custom-designed guide RNAs (gRNAs), previously published or commercially available gRNAs. For each target, multiple gRNAs were screened, and cleavage efficiency was assessed on day +7 using a T7 endonuclease assay (Figure 3D). The most efficient gRNA for each target was selected for subsequent KO experiments.

### AdCAR-T cell stimulation induces the upregulation of ICRs

To evaluate the expression dynamics of ICRs on AdCAR-T cells following activation, AdCAR-T^RNP Mock^, AdCAR-T^PD-1 KO^, and AdCAR-T^TIGIT KO^ were co-cultured with Raji cells in the presence of LLE-CD19 mAb and analyzed by flow cytometry at 0, 48, and 72 h (Figure 3E). CD96 expression remained stable across all conditions, indicating no response to stimulation. In contrast, CD276 showed a modest upregulation of less than 10% in all variants. The highest levels of ICR expression were observed at 72 h post-co-culture, with expression frequencies reaching 31% for PD-1, 33% for LAG-3, 18% for TIM-3, and 12% for TIGIT. A CAR-dependent upregulation was evident for PD-1, LAG-3, and TIM-3, while TIGIT expression appeared to be regulated independently of CAR stimulation. As expected, no upregulation of the respective target ICRs was detected in the KO cell lines, confirming efficient gene disruption. These results demonstrate that ICR expression is dynamically modulated following CAR activation and confirm the effective disruption of target genes in the KO models.

### TIGIT-KO enhances the antitumoral activity of AdCAR-T in AML

**Figure 4.**
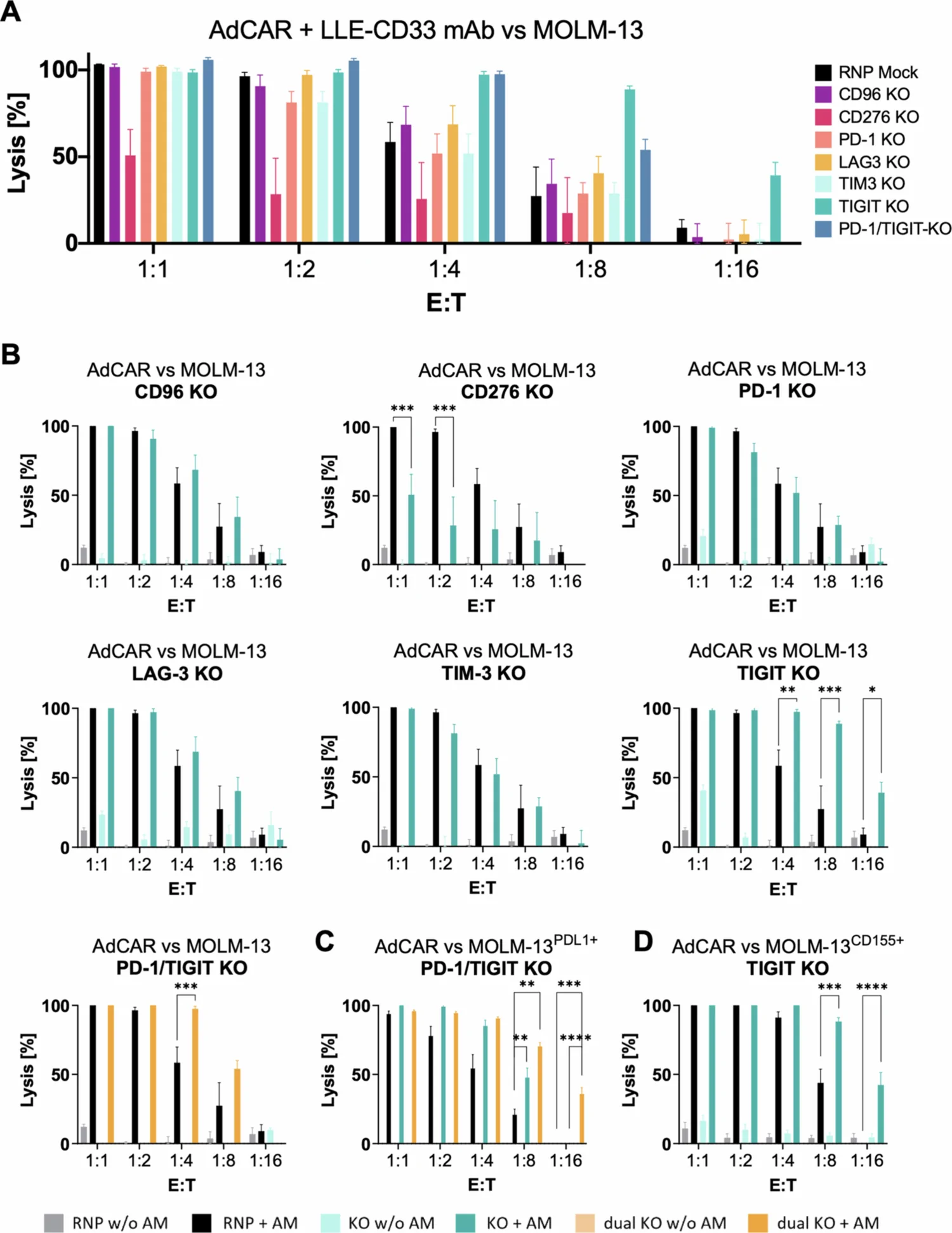
Functional evaluation of AdCAR-T cells with different ICR KOs. Target cell lysis was assessed using LCAs. (A) AdCAR-T cells with ICR KOs were co-cultured with MOLM-13 at the indicated E:T ratios for 72 h in the presence of 10 ng/mL LLE-CD33 mAb. (B) AdCAR-T cells with ICR KOs were co-cultured with MOLM-13 at the indicated E:T ratios for 72 h, in the presence or absence of 10 ng/mL LLE-CD33 mAb. (C) AdCAR-T^PD1-KO^ and AdCAR-T^PD1/TIGIT-KO^ cells were co-cultured with a MOLM-13 cell line overexpressing PDL1 (MOLM-13^PDL1+^) under the same conditions. (D) AdCAR-T^TIGIT-KO^ cells were co-cultured with a MOLM-13 cell line overexpressing CD155 (MOLM-13^CD155+^) under the same conditions. Data in panels A–D represent the mean ± SEM. *n* = 3 donors, with 3-6 technical replicates each. Statistical comparisons were performed within each fixed experimental condition (same target cell line, same E:T ratio, same time point). *n* refers to independent donors; technical replicates were summarized within donors. For comparisons involving more than two groups, one-way ANOVA with Tukey’s post hoc test was used, with *p*-values < 0.05 considered statistically significant. Only statistically significant differences are indicated. *p < 0.05; **p < 0.01; ***p < 0.001; ****p < 0.0001. Abbreviations: ICR: immune checkpoint receptor; LCA: luciferase-based cytotoxicity assay; E:T: effector-to-target ratio; SEM: standard error of the mean.

To investigate the antitumor efficacy of AdCAR-T cells with individual or combined ICR KOs, cytotoxic activity was evaluated using LCAs. AdCAR-T cells and their respective KO variants were co-cultured with the AML cell line MOLM-13 in the presence of the LLE-CD33 mAb to enable specific target recognition. All KO-modified AdCAR-T cells demonstrated efficient lysis of MOLM-13 cells, even at low E:T ratios of 1:4 (Figure 4A). This cytolytic response was strictly adapter-dependent, as no target cell killing was observed in the absence of LLE-CD33 mAb (Figure 4B). No statistically significant differences in cytotoxicity were observed between the AdCAR-T^RNP Mock^, AdCAR-T^CD96 KO^, AdCAR-T^PD-1 KO^, AdCAR-T^LAG-3 KO^, and AdCAR-T^TIM-3 KO^ conditions. CD276 KO led to a significant reduction in the cytolytic activity of AdCAR-T cells compared to the AdCAR-T^RNP Mock^ control. In contrast, TIGIT KO resulted in a significant increase in target cell lysis, particularly at low E:T ratios ranging from 1:4 to 1:16. Dual KO of PD-1 and TIGIT resulted in improved cytotoxicity at an E:T ratio of 1:4. To further investigate the impact of ICL-mediated inhibition, MOLM-13 cells were engineered to overexpress PDL1 or CD155 via lentiviral transduction (Figure S2A). Functional testing revealed that AdCAR-T^PD-1 KO + TIGIT KO^ exhibited significantly enhanced cytolytic activity against MOLM-13^PDL1+^ (Figure 4C), while AdCAR-T^TIGIT KO^ showed significantly increased cytolysis against MOLM-13^CD155+^ compared to AdCAR-T^RNP Mock^ (Figure 4D). These results highlight the distinct functional roles of individual ICRs in modulating AdCAR-T cell cytotoxicity and identify TIGIT, alone or in combination with PD-1, as a promising target to enhance antitumor efficacy in AdCAR-T cell therapy.

### Immune checkpoint blockade by mAbs matches or surpasses AdCAR-T^KO^ cell lysis

**Figure 5.**
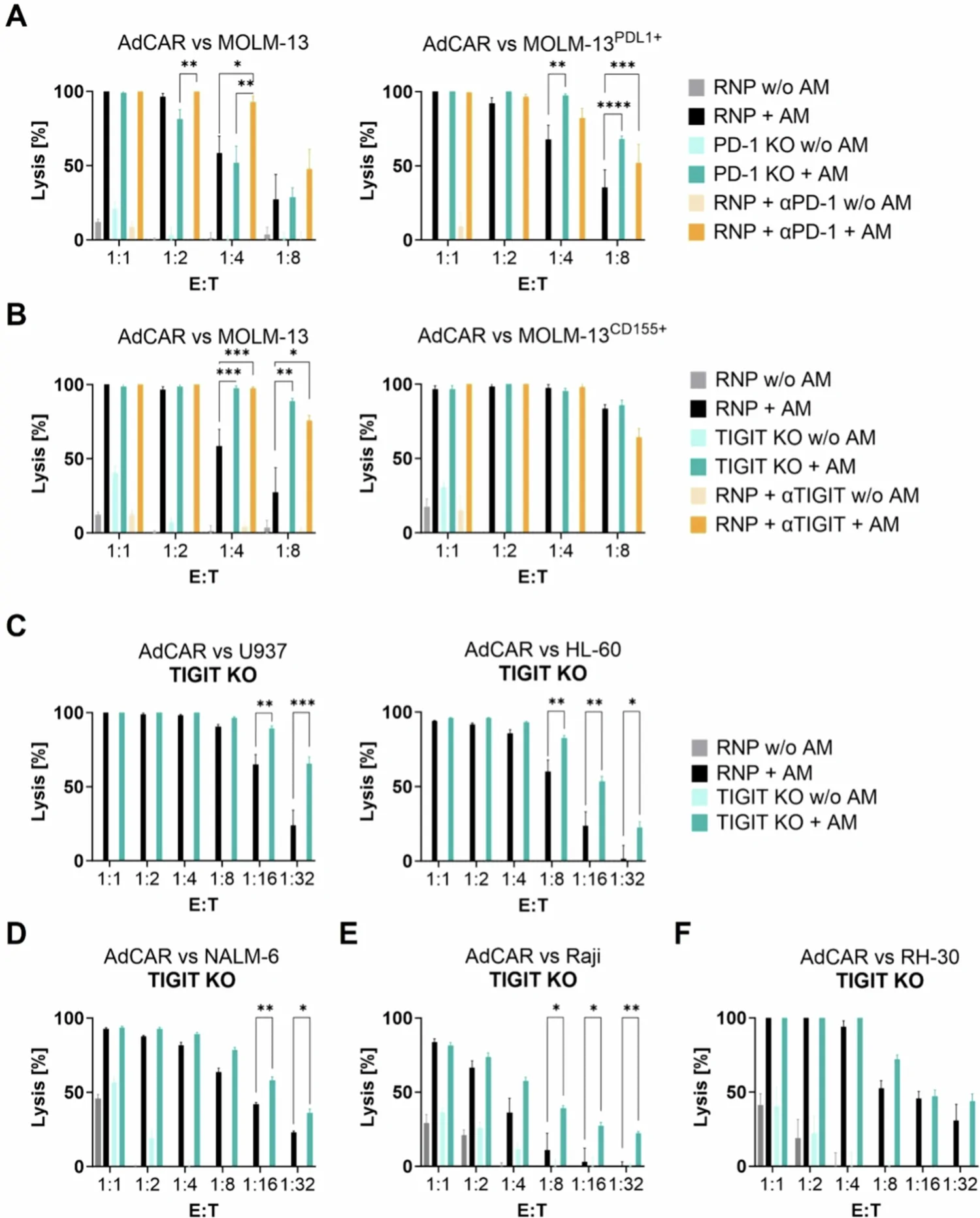
Functional evaluation of assorted AdCAR-T cell KOs against different tumor cell lines. (A) AdCAR-T^RNP Mock^ (RNP), AdCAR-T^PD-1 KO^ (PD-1 KO), and AdCAR-T^RNP Mock^ in combination with pembrolizumab (αPD-1) were co-cultured with MOLM-13 or the PDL1 overexpressing cell line MOLM-13^PDL1+^ at indicated E:T ratios for 72 h, in the presence or absence of 10 ng/mL LLE-CD33 mAb. *n* = 3 donors, with 3–5 technical replicates each. (B) AdCAR-T^RNP Mock^ (RNP), AdCAR-T^TIGIT KO^ (TIGIT KO), and AdCAR-T^RNP Mock^ in combination with tiragolumab biosimilar (αTIGIT) were co-cultured with MOLM-13 or the CD155 overexpressing cell line MOLM-13^CD155+^at the indicated E:T ratios for 72 h, in the presence or absence of 10 ng/mL LLE-CD33mAb. *n* = 3 donors, with 3-6 technical replicates each. (C) AdCAR-T^RNP Mock^ (RNP) and AdCAR-T^TIGIT KO^ (TIGIT KO) were co-cultured with the AML cell lines U937 and HL-60 at the indicated E:T ratios for 72 h or 48 h, respectively, in the presence or absence of 10  ng/mL LLE-CD33 mAb. *n* = 3 donors, with 6 technical replicates each. (D) AdCAR-T^RNP Mock^ (RNP) and AdCAR-T^TIGIT KO^ (TIGIT KO) were co-cultured with the B-cell precursor cell line NALM-6 at the indicated E:T ratios for 48 h, in the presence or absence of 10 ng/mL LLE-CD19 mAb. *n* = 3 donors, 5 technical replicates each. (E) AdCAR-T^RNP Mock^ and AdCAR-T^TIGIT KO^ were co-cultured with the Burkitt lymphoma cell line Raji at the indicated E:T ratios for 48 h with or without 10  ng/mL LLE-CD19 mAb. *N* = 3, 4 technical replicates each. (F) AdCAR-T^RNP Mock^ and AdCAR-T^TIGIT KO^ were co-cultured with the rhabdomyosarcoma cell line RH30 at indicated E:T ratios for 72 h, in the presence or absence of 10  ng/mL LLE-CD276 mAb. *N* = 3, 4 technical replicates each. Data shown in panels A–F represent mean ± SEM. Statistical comparisons were performed within each fixed experimental condition (same target cell line, same E:T ratio, same time point). *n* refers to independent donors; technical replicates were summarized within donors. For comparisons involving more than two groups, one-way ANOVA with Tukey’s post-hoc test was used, with *p*-values < 0.05 considered statistically significant. Only statistically significant differences are indicated. *p < 0.05; **p < 0.01; ***p < 0.001; ****p < 0.0001. Abbreviations: KO: knockout; LCA: luciferase-based cytotoxicity assay; E:T: effector-to-target ratio; SEM: standard error of the mean; RNP: ribonucleoprotein.

Clinically approved ICIs may represent a viable combinatorial approach with AdCAR-T cell products for therapeutic applications. Therefore, we evaluated the cytotoxic activity of AdCAR-T^PD-1 KO^ and AdCAR-T^TIGIT KO^ variants in comparison to the AdCAR-T^RNP Mock^ in combination with the clinically approved PD-1 blocking antibody pembrolizumab or a TIGIT-blocking biosimilar of tiragolumab. Cytotoxicity was measured using LCAs (Figure 5). Targeting MOLM-13 wildtype cells, AdCAR-T^RNP Mock^ with pembrolizumab showed significantly enhanced lysis compared to both AdCAR-T^PD-1 KO^ and AdCAR-T^RNP Mock^ at an E:T ratio of 1:4 (Figure 5A). Against MOLM13^PDL1+^ cells, AdCAR-T^PD1-KO^ demonstrated significantly enhanced cytolysis compared to AdCAR-T^RNP Mock^, whereas no significant difference was observed between AdCAR-T^PD1-KO^ and AdCAR-T^RNP Mock^ treated with pembrolizumab. Similarly, AdCAR-T^TIGIT KO^ and AdCAR-T^RNP Mock^ combined with tiragolumab biosimilar induced significantly enhanced lysis of MOLM-13 wild-type cells compared to AdCAR-T^RNP Mock^ (Figure 5B). When targeting MOLM-13^CD155+^ cells, no significant differences were observed between the groups. These results indicate that PD-1 and TIGIT blockade, via ICIs or gene editing, can enhance AdCAR-T cell cytotoxicity in a context-dependent manner.

### AdCAR-T^TIGIT KO^ demonstrates enhanced target cell lysis across multiple tumor models and target antigens

Based on the enhanced cytotoxicity of AdCAR-T^TIGIT KO^ against the AML cell line MOLM-13, we extended our analysis to additional tumor models using LCA (Figure 5C–F). AdCAR-T^TIGIT KO^ demonstrated significantly enhanced target cell lysis compared to AdCAR-T^RNP Mock^ against the AML cell lines U937 and HL-60, particularly at low E:T ratios, using an LLE-CD33 mAb as AM. Enhanced cytotoxicity was observed against the B-lineage-derived cell lines NALM-6 and Raji in the presence of LLE-CD19. In contrast, no significant differences in cytolytic activity were detected between AdCAR-T^TIGIT-KO^ and AdCAR-T^RNP Mock^ against the rhabdomyosarcoma cell line RH30 using LLE-CD276 mAb as AM. These results suggest that TIGIT KO enhances AdCAR-T cell cytotoxicity in a tumor type-dependent manner.

Additionally, AdCAR-T^TIGIT KO^ demonstrated significantly increased cytotoxicity against MOLM-13 when redirected via alternative adapter molecules, including LLE-CD33 mAb, LLE-CD38 mAb, LLE-CD135 mAb, or a combination of all three (Figure S6). These findings confirm the multitargeting capability of AdCAR-T^TIGIT KO^ and illustrate their functional adaptability across various antigen settings.

In summary, our results demonstrate pronounced intra- and inter-individual heterogeneity in ICL expression across tumor entities and patient-derived AML samples and show that both ICL and ICR expression are dynamically regulated in response to AdCAR-T cell engagement. Furthermore, targeting TIGIT through genetic KO or antibody-mediated blockade consistently improves AdCAR-T cell cytotoxicity in various tumor models.

## Discussion

CAR-T cell therapy has demonstrated therapeutic efficacy in AML, although at doses tenfold higher than those typically used in B-cell precursor-ALL.^16^ ^,^ ^17^ A major limitation is posed by the substantial hematotoxicity associated with CD33-targeted CAR-T cells, arising from on-target off-tumor effects.^17^ To overcome severe hematotoxicity, several strategies have been explored, including target gene inactivation or epitope editing in autologous hematopoietic stem cells to rescue hematopoiesis from CD33-, CD45-, or FLT3 (CD135)-targeted CAR-T cells.^19^ ^,^ ^20^ ^,^ ^41^ Additionally, fewer hematotoxic targets were identified and utilized, such as GRP78, to spare HSPCs.^42^ In line with the approaches of other research groups, we have previously developed the AdCAR technology to enable transient, multitargeted immunotherapy in AML, addressing key challenges such as on-target off-tumor toxicity and antigen heterogeneity.^23^ ^,^ ^43^

We confirmed that antileukemic CAR-T cell activity dynamically modulates the expression of ICL on AML cell lines, characterized by a marked upregulation of PDL1 and HLA-DR, both of which are known to be inducible by interferon-gamma.^44-46^ In contrast, CD155 expression was found to be downregulated. These dynamic alterations in ICL expression have functional implications for the role of ICRs in modulating antitumor activity. Specifically, elevated PDL1 expression in AML has been associated with reduced survival probability.^47^

Consistent with previous reports, antileukemic activity was associated with the upregulation of PD-1, TIM-3, and LAG-3 expression on T cells, mediated through T-cell receptor or CAR receptor engagement and signaling.^48-51^ Additionally, TIM-3 expression can be induced in an antigen-independent manner by common *γ*-chain cytokines.^52^

TIGIT was first identified as an ICR in 2009.^53^ In the present study, we demonstrate that the TIGIT-CD155 interaction plays a critical role in modulating AdCAR-T cell function in AML. Using CRISPR/Cas9-edited CAR-T cells, we show that both genetic inactivation of TIGIT and pharmacological TIGIT-blockade using a tiragolumab biosimilar antibody significantly enhance the antileukemic activity of AdCAR-T in AML. Additionally, the inhibitory impact of PD-1 in AML was demonstrated by the enhanced cytotoxicity of AdCAR-T cells following PD-1 KO, PD-1 receptor blockade using pembrolizumab, and dual KO of both PD-1 and TIGIT genes.^54^

In this study, we demonstrated that TIGIT KO enhances the cytotoxicity of AdCAR-T cells in CD155-positive AML and B-lineage-derived cancer cell lines (NALM-6 and Raji) but had no significant effect in the rhabdomyosarcoma cell line RH30, despite its high CD155 expression. This context-dependent effect suggests that the functional outcome of the TIGIT–CD155 axis is influenced by the competitive interplay with the activating receptor DNAM-1, and alternative co-stimulatory pathways for TIGIT, like NKG2D.^55^ DNAM-1 and NKG2D were highly expressed in AdCAR-T (Figure S2B) and thus are assumed to influence the antitumoral activity. TIGIT KO did not appear to affect DNAM-1 expression (Figure S7). Although RH30 might theoretically represent an ideal model due to its high CD155 expression, CD155 serves as a shared ligand for both inhibitory (TIGIT, CD96, and CRTAM) and activating (DNAM-1) receptors, making it difficult to predict the net immunologic effect.^55^ The influence of TIGIT versus DNAM-1 signaling depends on the co-expression profiles of both the ligands and the receptors, as well as on PD-1 co-expression.^56^ ^,^ ^57^ Importantly, both the proportion of ligand-expressing cells and the level of expression (represented by the MFIR) critically influence receptor engagement. Given the variability in ICL expression across and within tumor types, dissecting the relative contribution of individual pathways remains complex. These findings underscore the need for future studies to further delineate the contribution of individual receptors and signaling pathways, while also taking tumor-specific immune landscapes into account when evaluating the functional relevance of ICRs.^58^

Despite their ICLs being expressed at relevant levels, KO of the ICRs CD96, CD276, TIM-3, and LAG-3 did not result in enhanced antileukemic activity of AdCAR-T cells. Notably, therapies targeting LAG-3 and TIM-3 are currently under investigation in AML. These include the AARON trial (NCT04913922), which is evaluating LAG-3 blockade in relapsed or refractory AML, and the STIMULUS-AML2 trial (NCT04623216), which is assessing sabatolimab, a TIM-3-blocking antibody, in patients with posttransplant persistent minimal residual disease.^59^ ^,^ ^60^ Preclinical work by Zhang et al. has demonstrated improved CAR-T cell function in Raji cells following LAG-3 inactivation.^61^ Moreover, TIM-3-CD28 switch receptors have been designed to convert inhibitory TIM-3 signaling into an activating signal, leveraging TIM-3 overexpression in leukemias to enhance CAR-mediated antigen-specific activity.^62^ Notably, TIM-3 not only functions as an ICR on T cells but might also serve as a viable target antigen in AML, as highlighted in several recent studies.^15^ ^,^ ^63^ ^,^ ^64^

In our *in vitro* study, TIM-3 KO did not have any detectable impact on AdCAR-T cell function. However, culture conditions may significantly influence the experimental readout, and no relevant surface expression of galectin-9 was observed on any of the tested cancer cell lines. Galectin-9 acts as an autocrine ligand for TIM-3. TIM-3 and galectin-9 are both frequently overexpressed in AML, where they have been associated with unfavorable survival outcomes, especially in self-renewing leukemic stem cells (LSCs).^15^ ^,^ ^65^ ^,^ ^66^ In LSCs, the interaction of TIM-3 and galectin-9, along with protein kinase HCK and p120-catenin, predominantly activates the canonical WNT pathway, regulating cell proliferation and self-renewal.^48^ ^,^ ^67^ In AML, TIM-3 signaling has been shown to promote disease progression and treatment resistance through WNT pathway activation, whereas it promotes terminal exhaustion and apoptosis in T cells. Therefore, TIM-3 blockade has been proposed as a dual antileukemic strategy in AML, with the potential to simultaneously reduce T cell exhaustion and impair AML progression.^68^ ^,^ ^69^ Despite this mechanistic rationale, such effects were not observed in our *in vitro* model, possibly due to the absence of galectin-9 expression. Moreover, the functional relevance of TIM-3 is thought to become most apparent under conditions of chronic or repetitive antigen stimulation. Short-term LCAs with single-round antigen exposure, as performed in the present study, may therefore underestimate their contribution to AdCAR-T cell dysfunction.

Our findings show that CD276 KO significantly impaired the cytotoxic activity of AdCAR-T cells in AML. While CD276 has previously been reported to suppress CAR-T cell function in esophageal cancer, its biological role appears to be highly context-dependent.^70^ Both co-inhibitory and co-stimulatory effects of CD276 have been described, depending on the tumor entity and the cellular compartment in which it is expressed.^71^ ^,^ ^72^ In contrast, CD96 KO did not affect AdCAR-T cell function in our study. Notably, previous studies have shown that disruption of the CD96 signaling pathway, either through blocking antibodies or gene KO, can enhance the efficacy of EpCAM-targeted CAR-T cells in a preclinical colorectal cancer model, more effectively than PD-1 blockade.^73^ These discrepancies may result from tumor-specific differences in microenvironmental factors, ligand expression, and co-signaling dynamics, as well as from potential unintended effects of gene KOs on intracellular signaling pathways critical for CAR-T cell function.

While cancer cell ICL expression is commonly used to justify ICI treatment, clinical benefits have even been observed in patients lacking detectable ICL expression.^74^ This phenomenon can be attributed to the inhibitory effects mediated by healthy immune cells in the TME, such as tumor-associated macrophages (TAMs), which also express PDL1 and are therefore included in the evaluation of PDL1 immune scores.^75^ These controversially discussed findings highlight the complexity of the TME and the multitude of factors influencing the response to ICI therapy. Consequently, enhancing immune cell function by overcoming inhibitory signals within the TME has been shown to improve tumor control and prolong survival in both solid cancers and AML.^26-28^ ^,^ ^59^ ^,^ ^60^ Yet, therapeutic success depends on a combination of factors rather than a single dominant pathway.

A major limitation of both ICI monotherapy and its combination with CAR-T cell therapy remains the risk of severe immune-related adverse events, including hyperinflammatory responses affecting the skin, gastrointestinal tract, endocrine organs, and lungs, thereby restricting its applicability in a relevant number of patients.^76-78^ Therefore, genetic KO of ICRs in effector cells represents a promising strategy to reduce the risk of autoimmune toxicities commonly associated with ICI treatment.

While our study provides valuable insights into the role of ICRs in modulating AdCAR-T cell function, several limitations should be acknowledged. First, all functional assays were conducted *in vitro*, which does not fully recapitulate the complexity of the *in vivo* TME. Critical modulatory components such as myeloid-derived suppressor cells, regulatory T cells, stromal interactions, and cytokine gradients are absent in this setting and may substantially influence the efficacy and persistence of CAR-T cells. Secondly, although we demonstrated effective gene KO using CRISPR/Cas9, off-target effects or unintended disruptions of signaling networks cannot be entirely ruled out and warrant further genomic validation. Third, ICL and ICR expression patterns were assessed at fixed time points; however, these molecules are known to be dynamically regulated and context-dependent. Longitudinal analyses and *in vivo* models will be essential to better understand their kinetics and functional impact under therapeutic conditions.

Overall, the introduction of genetic modifications into highly potent and tightly regulated immune effector cells like AdCAR-T cells presents a promising strategy to overcome current limitations in cancer immunotherapy.

## Conclusion

Our results highlight the therapeutic potential of integrating ICR KOs into adapter-controlled CAR-T-cell systems and identify TIGIT as a promising candidate for further investigation, while the findings should be interpreted in light of the exploratory nature of this *in vitro* study. Both genetic and pharmacologic TIGIT inhibition enhanced antitumor efficacy in a context-dependent manner. This strategy not only circumvents the systemic toxicities associated with immune checkpoint blockade but also enables transient and combinatorial targeting through the AdCAR platform. Future work should validate these findings in *in vivo* models and evaluate the clinical feasibility and safety of integrating ICR KO into adapter-controlled CAR-T cell therapies for AML and other hematologic malignancies.

## Supplementary Material

Supplementary Material 20260619_Supplement_Oncoimmunology_REVISION_FINAL_updated (Clean).docx

## Data Availability

Original data are available upon request from the corresponding author, Patrick Schlegel (Patrick.Schlegel@ukr.de).

## Abbreviations

AdCAR: Adapter CAR
alloHSCT: allogeneic Hematopoietic Stem Cell Transplantation
AM: Adapter Molecule
AML: Acute Myeloid Leukemia
ATCC: American Type Culture Collection
CAR: Chimeric Antigen Receptor
Cas9: Bacterial Endonuclease
CRISPR: Clustered Regularly Interspaced Short Palindromic Repeats
CRTAM: Class I-Restricted T-Cell-Associated Molecule
CTLA-4: Cytotoxic T-Lymphocyte-Associated Protein 4
FcR: Fragment crystallizable Receptor
gRNA: guide Ribonucleic Acid
HSC: Hematopoietic Stem Cells
HPC: Hematopoietic Progenitor Cells
ICI: Immune Checkpoint Inhibition
ICL: Immune Checkpoint Ligand
ICR: Immune Checkpoint Receptor
KO: Knockout
LAG3: Lymphocyte-Activation Gene 3
LLE: Linker-Label-Epitope
mAb: monoclonal Antibody
MFIR: Median Fluorescence Intensity Ratio
PBMC: Peripheral Blood Mononuclear Cells
PD-1: Programmed cell Death protein 1
PDL1: Programmed Death-Ligand 1
PDL2: Programmed Death-Ligand 2
TcR: T-cell Receptor
TRAC: T-cell Receptor A Constant
TME: Tumor Microenvironment
TIGIT: T cell immunoreceptor with Ig and ITIM domains
TIM-3: T cell Immunoglobulin and Mucin domain-containing protein 3
WNT: Wingless-related Integration site
