# Supplementary material for: Immune checkpoint knockout screen reveals TIGIT-CD155 interaction as a significant modulator of adapter CAR-T cell function in acute myeloid leukemia
Source: Oncoimmunology. 2026 Sep 17;15(1):2717853. doi: 10.1080/2162402X.2026.2717853 (PMC13596916; doi:10.1080/2162402X.2026.2717853)
Supplement: Supplementary file 1 [file KONI_A_2717853_SM7923.docx]

**Supplementary data**

***Supplementary methods***

**The AdCAR and CD19-CAR architecture**

The designs of both CAR constructs are depicted in **Figure S1**. The AdCAR construct was empirically selected through systematic CAR establishment and optimization (22). It was designed in a 3^rd^-generation CAR format, incorporating a single-chain variable fragment (scFv) derived from the anti-LLE-mAb clone Bio2 4G10, arranged in a VH-VL configuration (variable light/variable heavy), consistent with our previous work (23). The CD19-CAR was designed in a 2^nd^-generation CAR format, employing an scFv derived from the 4G7 clone in a VL–VH configuration.

**Production of Lentivirus**

Lentivirus (LV) was generated as previously described (22). Viral supernatant was harvested, concentrated, and stored at -80°C according to the manufacturer´s instructions (TakaRa Lenti-X Concentrator). LV used for AdCAR-T cell production was provided by Miltenyi Biotec.

**Manufacturing of antibodies**

Monoclonal antibodies targeting CD19 (clone 4G7), CD33 (clone hP67.6, gemtuzumab), and TIGIT (tiragolumab-based silenced IgG1/kappa VH/VL) were produced using the ExpiCHO^TM^ transient expression system (ThermoFisher Scientific). Respective VH and LH sequences were cloned into the pcDNA^TM^3.1^(+)^ mammalian expression vector containing an Fc-attenuated IgG1 backbone with L234A/L235A substitutions. Transfections of ExpiCHO-S^TM^ cells were performed following the manufacturer´s Max titer protocol (ThermoFisher Scientific). Produced antibodies were subsequently purified using rProtein A GraviTrap columns (Cytiva), followed by desalting using PD10 Sephadex G25 columns (Cytiva). Antibody purity was evaluated through SDS Gel and liquid chromatography-mass spectrometry (LC-MS).

The VH and VL amino acid sequences of tiragolumab biosimilar were identical to those of tiragolumab (US20170088613A1). The antibody was produced according to the above-described protocol, and functional binding to target cells was confirmed after purification. To assess TIGIT binding, CD19-CAR-T cells were co-cultured with Raji cells at an E:T ratio of 1:2 in the presence of biotinylated tiragolumab biosimilar in a dilution series from 0 ng/mL to 1000 ng/mL. Biotinylated rituximab (anti-CD20 mAb) served as a control. After 48 hours of incubation, samples were stained with CD3-PE and anti-biotin-APC and analyzed by flow cytometry to quantify TIGIT binding efficiency (**Figure S5**).

**Antibody biotinylation**

Biotinylation was performed at 21°C for 1 hour in PBS buffer, employing a 3-fold molar excess of biotin-LC-LC-NHS (ThermoFisher Scientific), followed by separation of labeled antibodies from unreacted biotin using a Sephadex G-25 column (Cytiva) at antibody concentrations ranging from 1 to 10 mg/mL. Protein concentration was measured by absorption at 280 nm. Biotinylation was confirmed by LC-MS or flow cytometry using antigen-expressing cell lines and secondary detection with a fluorophore-conjugated anti-biotin antibody (Miltenyi Biotec). Antigen expression in these cell lines was previously confirmed with commercial antibodies of the same specificity.

**Generation of luciferase-expressing cell lines**

Luciferase-expressing cell lines were generated using a 3^rd^ generation lentiviral vector plasmid (Plasmid #104833; Addgene), which contains eFFly luciferase and the mCherry fluorescent protein. To enable surface detection, a truncated CD19 construct was cloned into the plasmid, replacing mCherry. Vector was produced as described above. Cell lines were transduced at a multiplicity of infection (MOI) ranging from 3 to 10. Transgene expression was confirmed by flow cytometry using anti-CD19-PE staining, and transduced populations were enriched via bulk fluorescence-activated cell sorting (FACS).

**Production of PDL1 and CD155 overexpressing cell lines**

Full-length human CD155 (Uniprot P15151-1) and PDL1 (Uniprot Q9NZQ7-1) were stably introduced into MOLM-13 by lentiviral transduction. Coding sequences were cloned into a 3^rd^ generation lentiviral vector under the control of the constitutive EF1α promoter, and lentiviral particles were produced as described above. MOLM-13 cells were transduced at an MOI of 3, followed by bulk enrichment of transduced cells using FACS. Post-sort expression levels of CD155 and PDL1 are shown in **Figure S1A**.

**Establishment of CRISPR/Cas9 mediated KO in CAR-T expansion protocol**

To determine the optimal time point for CRISPR/Cas9-based gene KOs within the CAR-T cell manufacturing process, PBMCs were isolated from whole-blood samples obtained from healthy donors using a Ficoll-Paque density gradient centrifugation (Biocoll, Sigma-Aldrich®). T cells were subsequently purified via magnetic cell separation using anti-CD4 and anti-CD8 microbeads, following the manufacturer's instructions (Miltenyi Biotec). For each condition, 800,000 primary T cells were seeded. For transfection of 0.8 x 10^6^ cells, 60 pmol sgRNA and 60 pmol TrueCut Cas9 Protein v2 (ThermoFisher Scientific) were used. Electroporation was performed at various time points, ranging from day -2 to day +4, relative to TransAct^TM^ stimulation on day 0 and lentiviral transduction on day +2. Cells were cultured at a 0.5 to 1 x 10^6^ cells/mL density in supplemented TexMACS^TM^ medium.

On day +10, cell counts were determined using a Neubauer counting chamber, while KO efficiency, viability (via propidium iodide (PI) exclusion staining), and transduction efficiency were assessed via flow cytometry. On day +12, cells were cryopreserved according to standard operating procedures, using cryopreservation medium (10% DMSO, 90% FBS), and stored in liquid nitrogen. Prior to functional assessment, cells were thawed and cultured for 48 hours in TexMACS™ medium to ensure recovery and viability.

**Generation of AdCAR-T KOs using CRISPR/Cas9 technology**

Multiple gRNAs were screened for each specific gene KO. The gRNAs were either previously published, pre-designed by ThermoFisher Scientific, or custom-designed using the online tool “CHOPCHOP” (http://chopchop.cbu.uib.no/, accessed April 16, 2020), and then synthesized by ThermoFisher Scientific. Subsequently, the gRNAs were screened for their KO efficiency in primary T cells. A comprehensive list of used gRNAs is provided in **Table S3**. All gRNAs were dissolved in TRIS buffer (10 mM Tris, pH 8.0) at a concentration of 100 pmol/µl, aliquoted to prevent freeze-thaw cycles, and stored at -20°C. For transfection of 2 x 10^6^ cells, 75 pmol gRNA and 75 pmol TrueCut Cas9 Protein v2 (ThermoFisher Scientific) were used. To form the CRISPR/RNP complex, gRNA and Cas9 were mixed and incubated at room temperature for 10 to 15 minutes. Electroporation was carried out using the NEON^TM^ Transfection system 100 µl Kit (ThermoFisher Scientific) following the manufacturer´s instructions for T cells (1600 V, 3 pulses, buffer T). KOs were performed on day +3 of the expansion protocol. RNP controls were transfected with TrueCut Cas9 Protein v2 only. KO efficiency was determined on day +7 using the GeneArt™ Genomic Cleavage Detection Kit (ThermoFisher Scientific) according to the manufacturer´s instructions.

**Luciferase-Based Cytotoxicity Assays (LCA)**

LCA was performed as described before (22). In brief, tumor cells were seeded into white 96-well flat-bottom plates (Greiner Bio-One) at a density of 30,000 cells per well for suspension cell lines and 25,000 cells per well for adherent cell lines, in complete RPMI or DMEM media following ATCC recommendations. Adherent cells were plated 4 hours before adding effector cells to allow cell adherence to the plates. Effector cells were seeded at the specified E:T ratios, ranging from 1:1 to 1:32. Antibody concentration was maintained at a therapeutic level of 10 ng/mL, while synthetic D-luciferin (Sigma-Aldrich) was added at a final concentration of 4 µg/mL. Tiragolumab biosimilar was added at a concentration of 10 µg/mL, and pembrolizumab at a concentration of 1 µg/mL. The final volume per well was 200 µl. Plates were incubated at 37°C, 95% humidity, and 5% CO_2_. Bioluminescence was measured after 48 hours and 72 hours using the Tecan SPARK microplate reader (PerkinElmer). Target cell lysis was determined based on the relative luminescence of experimental conditions compared to a dilution series of standard controls.

**Primary pediatric AML samples**

Bone marrow samples were collected from pediatric AML patients at the time of initial diagnosis or relapse, as well as from healthy donors, following written informed consent obtained from their caregivers. This study was designed and conducted in accordance with the principles of the Declaration of Helsinki and was approved by the local Institutional Review Board (819/2017BO1, 674/2017BO2).

**Immunophenotyping of pediatric AML samples**

Immunophenotyping of primary pediatric AML and healthy donor bone marrow samples was conducted using a Cytek Aurora 5-laser spectral flow cytometer (Cytek Biosciences). Optimal dilutions for antibodies and viability dyes were determined through titration assays, using cell lines expressing the target antigen alongside antigen-negative controls. Patient´s bone marrow samples, initially stored in liquid nitrogen, were thawed and treated with DNase 1 before staining. 20 U/ml DNase 1 (ThermoFisher Scientific) was diluted in DPBS (pH 7.0, containing 0.5 mM MgCl_2_ and 1 mM CaCl_2_ (ThermoFisher Scientific)) and supplemented with 0.5% human albumin solution (HAS), following the manufacturer´s instructions. To prevent non-specific antibody binding to Fc receptors, cells were blocked with a human FcR blocking reagent (Miltenyi Biotec). Antibody staining was carried out at 4°C in CliniMACS® PBS/EDTA buffer (Miltenyi Biotec), supplemented with 0.5% HAS. Used antibodies and dilutions are listed in **Table S2**. Single stain reference controls were established using UltraComp eBeads^TM^ (ThermoFisher Scientific) and cells with confirmed antigen expression. The following cell lines were used: MOLM-13 wildtype for viability and CD33 expression; T cells for CD45 and CD3 expression; Stem cells for CD34 expression; MOLM-13^CD155+^ overexpression for CD155 expression; RH30 for PDL2 expression; MOLM-13^PDL1+^ overexpression of PDL1 expression; LS for CD276 expression; Raji for HLA-DR expression.

Samples were acquired and unmixed using the “Cytek Assay Settings” in SpectroFlo® V3.0.3 software. Subsequent data analysis was performed using FlowJo 10.8 software. Antigen positivity was evaluated by comparison with fluorescence minus one (FMO) controls. The MFIR was calculated by dividing the MFI of the sample by that of the corresponding FMO control.

**Data visualization and statistics**

Flow cytometric data were analyzed using FlowJo v10.8 (Becton Dickinson). Data visualization was performed using GraphPad Prism9 version 9.4.1 (238) (GraphPad Software Inc.). Schematic illustrations were generated using Biorender. Statistical comparisons were performed within each fixed experimental condition (same target cell line, same E:T ratio, same time point). n refers to independent donors; technical replicates were summarized within donors. For comparisons involving more than two groups, one-way ANOVA with Tukey’s post hoc test was used, with p-values < 0.05 considered statistically significant. Sample size (n) refers to independent healthy volunteer donors. All statistical analyses were conducted using GraphPad Prism software (version 9.4.1). Data are presented as mean ± SEM.

***Supplementary Materials***

**Supplementary Table 1**: Overview of cell lines and primary cells used in this study.

| **Cell line** | **Specification** | **Details** | **DSMZ No.** |
| --- | --- | --- | --- |
| MOLM-13 | Human AML-derived cell line | In suspension | ACC 554 |
| U937 | Human histiocytic lymphoma-derived monocyte-like cell line | In suspension | ACC 5 |
| HL-60 | Human AML-derived cell line | In suspension | ACC 3 |
| Raji | Human Burkitt lymphoma-derived cell line | In suspension | ACC 319 |
| JeKo-1 | Human B cell lymphoma-derived cell line | In suspension | ACC 553 |
| NALM-6 | Human B cell precursor leukemia-derived cell line | In suspension | ACC 128 |
| RH30 | Human rhabdomyosarcoma-derived cell line | Adherent | ACC 489 |
| NCI-H226 | Human lung squamous cell carcinoma-derived cell line | Adherent | CRL-5826 |
| HOP-92 | Human lung non-small cell carcinoma-derived cell line | Adherent | CVCL_1286 |
| A549 | Human lung squamous type carcinoma-derived cell line | Adherent | ACC 107 |
| DU-145 | Human prostate carcinoma-derived cell line | Adherent | ACC 261 |
| 22Rv1 | Human prostate carcinoma-derived cell line | Adherent | ACC 438 |
| PBMC | Human, isolated from whole blood from healthy donors | In suspension |  |
| Stem cells | Human, isolated from bone marrow of healthy donors | In suspension |  |
| T cells | Human, isolated from whole blood from healthy donors | In suspension |  |

***Supplementary Table 2****: Overview of flow cytometry antibodies used in this study.*

| **Antigen** | **Conjugate** | **Dilution** | **Clone** | **Supplier** | **Isotype** | **Cat. #** |
| --- | --- | --- | --- | --- | --- | --- |
| CD3 | VioBlue | 1/50 | REA613 | Miltenyi Biotec | recombinant human IgG1 | 130-114-519 |
| CD3 | Spark Blue™550 | 1/20 | SK7 | BioLegend® | Mouse IgG1, κ | 344851 |
| CD19 | APC | 1/50 | REA675 | Miltenyi Biotec | recombinant human IgG1 | 130-113-642 |
| CD19 | APC | 1/50 | REA675 | Miltenyi Biotec | recombinant human IgG1 | 130-113-642 |
| CD33 | APC | 1/50 | REA775 | Miltenyi Biotec | recombinant human IgG1 | 130-111-020 |
| CD33 | BUV563 | 1/25 | WM53 | BD Biosciences | Mouse BALB/c IgG1, κ | 741369 |
| CD34 | BV711 | 1/37 | 8G12 | BioLegend® | Mouse IgG1, κ | 745543 |
| CD38 | BV605 | 1/37 | HB-7 | BioLegend® | Mouse IgG1, κ | 356642 |
| CD45 | BB515 | 1/25 | HI30 | BD Biosciences | Mouse IgG1, κ | 564586 |
| CD96 | PE | 1/50 | REA 195 | Miltenyi Biotec | recombinant human IgG1 | 130-101-032 |
| CD112 | PE | 1/50 | REA1195 | Miltenyi Biotec | recombinant human IgG1 | 130-122-782 |
| CD112 | PE-Vio®615 | 1/50 | REA1195 | Miltenyi Biotec | recombinant human IgG1 | 130-122-784 |
| CD123 | APC-Fire^TM^ 810 | 1/37 | 6H6 | BioLegend® | Mouse IgG1, κ | 306053 |
| CD135 | PE-Cy^TM^ 5 | 1/10 | BV10A4H2 | BioLegend® | Mouse IgG1, κ | 313310 |
| CD155 | PE | 1/50 | REA1081 | Miltenyi Biotec | recombinant human IgG1 | 130-118-998 |
| CD155 | AF™700 | 1/25 | SKII.4 | BioLegend® | Mouse IgG1, κ | 337629 |
| CD226 | PE | 1/50 | REA1040 | Miltenyi | recombinant human IgG1 | 130-117-489 |
| CD273 | PE | 1/50 | REA985 | Miltenyi Biotec | recombinant human IgG1 | 130-116-563 |
| CD273 | BB700 | 1/10 | MIH18 | BD Biosciences | Mouse IgG1, κ | 746072 |
| CD274 | PE | 1/50 | REA1197 | Miltenyi Biotec | recombinant human IgG1 | 130-122-809 |
| CD274 | PE-Fire™ 810 | 1/10 | 29E.2A3 | BioLegend® | Mouse IgG2b, κ | 329755 |
| CD276 | PE | 1/50 | REA1094 | Miltenyi Biotec | recombinant human IgG1 | 130-118-570 |
| CD276 | APC-Vio®770 | 1/25 | REA1094 | Miltenyi Biotec | recombinant human IgG1 | 130-118-576 |
| CD279 | PE | 1/50 | REA1165 | Miltenyi Biotec | recombinant human IgG1 | 130-120-382 |
| CD371 | BUV737 | 1/25 | 50C1 | BD Biosciences | Mouse IgG2a, κ | 748888 |
| Galectin-9 | PE | 1/50 | REA435 | Miltenyi Biotec | recombinant human IgG1 | 130-124-237 |
| HLA-DR | PE | 1/50 | REA805 | Miltenyi Biotec | recombinant human IgG1 | 130-111-789 |
| HLA-DR | BV785 | 1/25 | L243 | BioLegend® | Mouse IgG2a, k | 307642 |
| IL1RAP | PE-Vio®770 | 1/50 | REA558 | Miltenyi Biotec | recombinant human IgG1 | 130-108-754 |
| Lag3/ CD223 | PE | 1/50 | REA351 | Miltenyi Biotec | recombinant human IgG1 | 130-120-470 |
| MIC A/B | AF^TM^ 647 | 1/20 | 6D4 | BioLegend® | Mouse IgG2a, k | 320914 |
| MSLN | PE | 1/50 | REA1057 | Miltenyi Biotec | recombinant human IgG1 | 130-118-095 |
| TIGIT | PE | 1/50 | REA1004 | Miltenyi Biotec | recombinant human IgG1 | 130-116-814 |
| TIM-3/ CD366 | PE | 1/50 | REA635 | Miltenyi Biotec | recombinant human IgG1 | 130-119-785 |
| TRAC | PE | 1/50 | IP26 | BD Biosciences | Mouse IgG1, k | 4202641 |

***Supplementary Table 3****: Overview of screened single guide RNAs (sgRNAs). sgRNAs selected for functional evaluation are indicated in bold.*

| Target | Internal no. | Sequence 5`🡪 3` | Designed by |
| --- | --- | --- | --- |
| **CD96** | **11** | **GGCACAGTAGAAGCCGTATT** | **Invitrogen™ TrueGuide™** |
| **CD276** | **40** | **TCTCCAGCACACGAAAGCCA** | **CHOPCHOP** |
| CD276 | 41 | CAGCCCCACGGTCACCCACA | CHOPCHOP |
| CD276 | 42 | GTGGTCACGTTGCCAGTCAG | Marson Lab (79) |
| **PD-1** | **27** | **CGTGTCACACAACTGCCCAA** | **Marson Lab** (79) |
| PD-1 | 3 | ACCGCCCAGACGACTGGCCA | Invitrogen™ TrueGuide™ |
| **LAG-3** | **47** | **GTTCCGGAACCAATGCACAG** | **Marson Lab** (79) |
| LAG-3 | 46 | GGGTGGCTCCAGGTAAAACG | CHOPCHOP |
| LAG-3 | 48 | GGGAGTTACCCAGAACAGTG | Marson Lab (79) |
| **TIM-3** | **50** | **GGCCCAGGTAACTATGCATG** | **CHOPCHOP** |
| TIM-3 | 49 | ATAGGCATCTACATCGGAGC | CHOPCHOP |
| TIM-3 | 51 | TGTGTTTGAATGTGGCAACG | Marson Lab (79) |
| **TIGIT** | **52** | **CAGGCACAATAGAAACAACG** | **CHOPCHOP** |
| TIGIT | 53 | CATCTGCACAGCAGTCATCG | Marson Lab (79) |
| **TRAC** | **58** | **TCAGGGTTCTGGATATCTGT** | **CHOPCHOP** |

***Supplementary Table 4****: Characteristics of analyzed primary pediatric AML specimen*

**Primary AML**	20

**Relapsed AML**	9

**Female patients**	11

**Male patients**	18

**Median age at diagnosis**	11.5

**FAB classification of tested samples**

M1	1

M2	9

M3	1

M4	5

M5	5

M7	1

MDS	2

Unclassified	5

***Supplementary Figure Legends***

**Figure S1. Architecture of the AdCAR and CD19 CAR constructs.** Schematic illustration of the AdCAR system and the conventional CD19 CAR design. **A** Structure of the third-generation AdCAR targeting CD33 via an anti-LLE scFv fused to an IgG4 hinge region. The intracellular signaling domains consist of CD28, 4-1BB, and CD3ζ. **B** Structure of the second-generation CD19 CAR, comprising an anti-CD19 scFv linked to CD8α hinge and transmembrane domains. The intracellular signaling domains consist of 4-1BB and CD3ζ. Abbreviations: AdCAR: adapter chimeric antigen receptor; LLE: linker-label-epitope; scFv: single-chain variable fragment.

**Figure S2.** **Characterization of engineered MOLM-13 cell lines and expression of activating immune receptors on assorted AdCAR-T cells. A** Flow cytometric analysis of MOLM-13 wildtype cells and the overexpression variants MOLM-13^PDL1+^ and MOLM-13^CD155+^. Enhanced surface expression of PDL1 and CD155 was achieved through lentiviral transduction. **B** Immune phenotyping of the activating immune receptors DNAM-1 and NKG2D in AdCAR-T^RNP Mock^ and AdCAR-T^TIGIT KO^, demonstrating a comparable expression profile across conditions. Abbreviations: AdCAR: adapter chimeric antigen receptor; RNP: ribonucleoprotein; KO: knockout; scFv: single-chain variable fragment.

**Figure S3. Schematic illustration of the gating strategy for the flow cytometric analysis of primary and relapsed pediatric AML samples and healthy donor specimens.** Cells were initially identified based on FSC and SSC properties. Doublets were excluded by comparing the area (FSC-A/SSC-A) and height (FSC-H/SSC-H) of both FSC and SSC signals. Viability was assessed using the amine-reactive viability dyes, allowing the exclusion of cells with compromised membrane integrity, indicative of early or late apoptosis, or necrotic damage. In primary pediatric AML samples, leukemic blasts were identified by their distinct morphology and size, as well as their characteristic downregulation of CD45 (CD45^dim^) compared to other bone marrow-derived cells. In healthy bone marrow samples (lower panel), the CD45 positive cell subsets are comprised of hematopoietic stem cells and progenitor cells (HSPCs and HSCs). HSPCs are further distinguished by the expression of the cyclic ADP hydrolase CD38 (CD34+CD38+), whereas pluripotent HSCs exhibited a CD34+CD38− phenotype, reflecting an earlier developmental stage. Abbreviations: AML: acute myeloid leukemia; FSC: forward scatter; SSC: side scatter; HSPC: hematopoietic stem and progenitor cell; HSC: hematopoietic stem cell.

**Figure S4.** **ICL expression in primary pediatric AML samples at initial diagnosis and relapse, as well as in relation to T cell infiltration in the bone marrow.** **A** Flow cytometric analysis of ICL expression on leukemic blasts from primary pediatric AML samples obtained at initial diagnosis and relapse. Expression levels are presented as the percentage of ICL-positive blasts. **B** Frequency of bone marrow infiltrating T cells in pediatric AML patients (AML, n=29) compared to the physiological CD45 intermediate population of healthy bone marrow controls (HD, n=5). **C** ICL expression on leukemic blasts stratified by the extent of T cell infiltration in the bone marrow: T_low_: 0-5% T cell infiltrate, T_medium_: 5-20% T cell infiltrate, and T_high_ > 20% T cell infiltrate. Data are presented as individual values with the mean indicated by a horizontal line. Statistical analysis was performed using one-way ANOVA with Tukey’s post hoc test; p-values < 0.05 were considered statistically significant. No statistically significant differences were observed between the groups. Abbreviations: AML: acute myeloid leukemia; ICL: immune checkpoint ligand; HD: healthy donor; ANOVA: analysis of variance.

**Figure S5**. **Evaluation of tiragolumab biosimilar binding specificity.** Second-generation CD19 CAR-T cells were co-cultured with MOLM-13 cells at an E:T ratio of 1:2 in duplicates. A dilution series of biotinylated tiragolumab biosimilar ranging from 0 ng to 1000 ng was introduced. Biotinylated rituximab (anti-CD20 mAb) served as a control. After 48 hours of incubation, samples were stained with CD3-PE and anti-Biotin-APC and analyzed by flow cytometry. T cells were identified by CD3 expression, and biotin-positive events were quantified within this population. Unstained controls were used to define gating thresholds. Data are shown as mean ± SEM. Abbreviations: CAR: chimeric antigen receptor; E:T: effector-to-target ratio; mAb: monoclonal antibody; SEM, standard error of the mean.

**Figure S6. Combinatorial antigen targeting of AdCAR-T cells with TIGIT KO. A** AdCAR-TRNP Mock (RNP) and AdCAR-TTIGIT KO (TIGIT KO) were co-cultured with MOLM-13 at the indicated E:T ratios for 72 hours, in the presence of different adapter molecules (LLE-CD33 mAb, LLE-CD38 mAb, or LLE-CD135 mAb) or a combination thereof. Each adapter molecule was used at a concentration of 10 ng/mL, the combinatorial condition contained 30 ng/mL total adapter molecule (10 ng/mL each). n=2, with 6 technical replicates each. **B-D** AdCAR-TRNP Mock (RNP) and AdCAR-TTIGIT KO (TIGIT KO) were co-cultured with MOLM-13 in the presence or absence of: **B** LLE-CD33 mAb, **C** LLE-38 mAb, and **D** LLE-135 mAb (all at 10 ng/mL) for 72 hours to assess individual target engagement. **E** AdCAR-TRNP Mock (RNP) and AdCAR-TTIGIT KO (TIGIT KO) were co-cultured with MOLM-13 at the indicated E:T ratios for 72 hours in the presence of a triple combination of LLE-CD33, LLE-CD38, and LLE-CD135 mAbs at a total concentration of 30 ng/mL (10 ng/mL each). Data in panels A–E are presented as mean ± SEM. Statistical comparisons were performed within each fixed experimental condition (same target cell line, same E:T ratio, same time point). n refers to independent donors; technical replicates were summarized within donors. For comparisons involving more than two groups, one-way ANOVA with Tukey’s post hoc test was used, with p-values < 0.05 considered statistically significant. Only statistically significant differences are indicated. *p<0.05; **p<0.01; ***p<0.001; ****p<0.0001. Abbreviations: AdCAR: adapter chimeric antigen receptor; KO: knockout; RNP: ribonucleoprotein; E:T: effector-to-target ratio; mAb: monoclonal antibody; SEM: standard error of the mean; ANOVA: analysis of variance.

**Figure S7. DNAM-1 (CD226) surface expression across all CAR-T ICR-KO variants. A** Representative histograms of DNAM-1 (PE-A) surface expression on AdCAR T cells following CRISPR/Cas9-mediated KO of the indicated ICR genes. The FMO control is shown in grey. The RNP Mock condition in black. **B** DNAM-1 expression on CD33-CAR T cells derived from three independent donors (D1-D3), comparing RNP Mock (red and TIGIT KO conditions. The FMO control is shown in grey. DNAM-1, DNAX accessory molecule-1; CAR, chimeric antigen receptor; FMO, fluorescence-minus-one; RNP, ribonucleoprotein; KO, knockout.

***Supplementary Figures***

**Figure S1**

**Figure S2**

**Figure S3**

**Figure S4**

**Figure S5**

**Figure S6**

**Figure S7**
